# A Cross-Sectional Survey of Labor Pain Control and Women’s Satisfaction

**DOI:** 10.3390/ijerph19031741

**Published:** 2022-02-03

**Authors:** Jakub Pietrzak, Wioletta Mędrzycka-Dąbrowska, Lucyna Tomaszek, Magdalena Emilia Grzybowska

**Affiliations:** 1 Department of Obstetrics and Gynecological Nursing, Medical University of Gdansk, 80-211 Gdańsk, Poland; jakub.pietrzak@gumed.edu.pl; 2 Department of Anesthesiology Nursing & Intensive Care, Medical University of Gdansk, 80-211 Gdańsk, Poland; 3 Faculty of Medicine and Health Sciences, Andrzej Frycz Modrzewski Krakow University, 30-705 Krakow, Poland; ltomaszek@igrabka.edu.pl; 4 Director of Nursing Band, National Institute of Tuberculosis and Lung Diseases, 34-700 Rabka-Zdrój, Poland; 5 Department of Gynecology, Gynecological Oncology and Gynecological Endocrinology, Medical University of Gdansk, 80-214 Gdańsk, Poland; mgrzybowska@gumed.edu.pl

**Keywords:** pain, delivery, pharmacological methods, non-pharmacological methods, level of hospital reference

## Abstract

**Introduction:** Pain experienced during labor is a symptom of contractile activity and is a physiological feature of the uterus that occurs at the appropriate stages of labor. For the majority of women, labor pain is the most severe pain they will ever experience, and therefore should be relieved. Objective: (1) To evaluate labor pain intensity before and after using non-pharmacological and pharmacological interventions; (2) to assess women’s satisfaction of labor pain management. Methods: A multicenter cross-sectional survey study was performed on 500 women who gave birth in different reference level hospitals (i.e., I, II, III). Pain intensity was assessed according the Numeric Rating Scale (range 0–10), whereas women’s satisfaction was measured with a 5 point Likert scale. Results: The use of both non-pharmacological (median 6.7 (5; 8) vs. 4.5 (3.3; 5.5)) and pharmacological methods (median 8 (7; 9) vs. 5 (3; 6)) resulted in a significant reduction in pain (*p* < 0.01). Water immersion and epidural anesthesia proved to be the most effective non-pharmacological and pharmacological methods, respectively. In hospitals of reference I, analgesic management was based primarily on the use of non-pharmacological techniques, less often mixed, i.e., non-pharmacological and pharmacological techniques (27.5%). On the other hand, in hospitals with higher referentiality, mixed methods were used more often (level II—65.8%; level III—81.2%). Pain intensity was significantly higher (*p* < 0.0001) in hospitals with reference level I (median 5 (4; 6)) than in the hospitals with reference level II (median 4.2 (3; 5)) or level III (median 4.2 (3.3; 5.4)). Epidural anesthesia was most often performed (60%) in the hospital of reference II. Women’s satisfaction (median 4 (3; 5)), inter alia, was associated with the effectiveness of applied methods. Conclusions: The study findings suggest that women giving birth in hospitals of higher referentiality have better control of labor pain due to access to pharmacological methods. Epidural anesthesia remains the gold standard for relieving labor pain. The choice of a specific method is determined by the degree of hospital and associated with the pain referentiality.

## 1. Introduction

Labor is a critical and important period in a woman’s life. However, most women suffer from high-intensity pain during labor [1]. The pain experienced during delivery is mainly a physiological symptom, but psychological or social factors are also involved in its perception [2,3]. Pain relief during delivery is desirable to reduce the mother’s suffering and accelerate the progress of labor. The reduction of pain intensity requires appropriate techniques, as most women wish to experience some pain level during delivery [1]. The results of studies demonstrate that more than 23% of primiparas characterize this pain as unbearable, 65% as very strong or strong, and only 9% as bearable. On the other hand, only 17% of multiparas describe this pain as unbearable, 46% as very strong or strong, and 25% as acceptable [3,4]. The factors which have an impact on labor pain are physiological, psychological, and social. The psychological factors refer to the approaches and beliefs concerning labor pain. Modern ideas and the impact of the Western approach to pain have gradually changed the perception and desire for pain relief during labor [5,6]. Labor pain relief requires teamwork, including the cooperation of the anesthetist, midwife, and obstetrician. When selecting the adequate analgetic technique for a woman in labor, one should bear in mind that the chosen method should be safe for both the mother and the child [7]. Appropriate analgesia is vital because pain elevates the level of circulating catecholamines, which impairs the perfusion of the uterus [1]. The selection of the analgetic technique depends on the patient’s condition, the progress of labor, and the available resources [2]. Pharmacological methods of labor pain relief include the administration of parenteral opioids, inhalation, and regional methods. Non-pharmacological pain management includes a broad spectrum of techniques, including water immersion (shower, bathtub) or transcutaneous electrostimulation. They aim to alleviate the physical perception of pain and prevent suffering by improving psychological and mental elements of care [4]. Pain management has a low priority in many countries with low and medium incomes [8]. Such an approach is mainly purely theoretical and includes racial differences or religious background, leading some women to believe that labor pain is “God’s will” [5,6]. Therefore, some authors emphasize that women’s preferences for pain relief methods during labor are not thoroughly investigated. The literature offers conflicting results about the choices of pain relief methods during labor. In Australia, 77% of women in labor use pharmacological intervention for pain relief, including regional analgesics (33%), whereas in some hospitals in the United States, as many as 80% of laboring women receive epidural analgesia. The partner’s preference and having a previous epidural were the two significant predictors of a woman receiving an epidural [6,9].

This study aimed to evaluate labor pain intensity before and after using non-pharmacological and pharmacological interventions in hospitals of the Pomeranian Voivodeship and the factors determining the patients’ satisfaction with pain management.

## 2. Materials and Methods

### 2.1. Study Design, Setting

**Figure 1:**
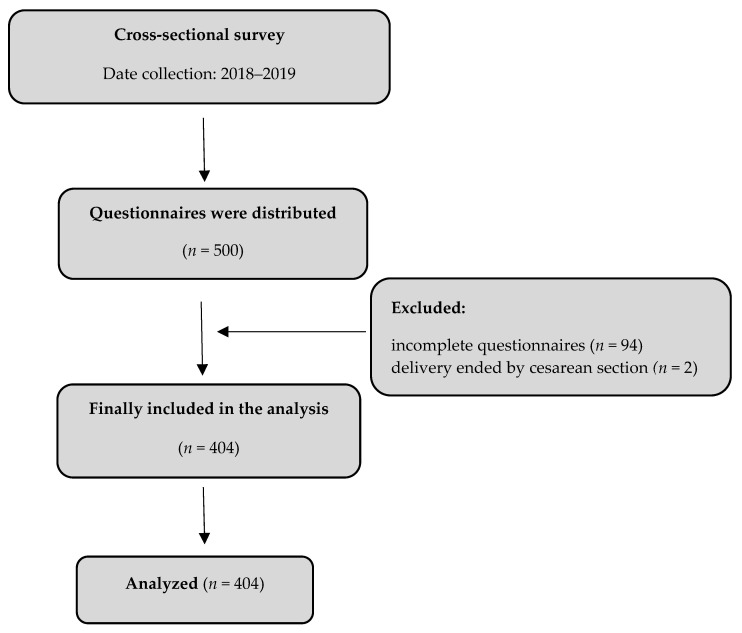
Flow diagram.

This was a cross sectional survey study carried out in 5 selected hospitals of different levels of care (in Poland—“levels of reference”) in the Pomeranian Voivodeship (geographic regions within Poland similar to a province or state). The project was implemented from 2018 to 2019, among 500 women (Figure 1). Consent was obtained from the management of each hospital and the heads of the departments of gynecology and obstetrics. Participation in the study was anonymous and voluntary. The patients received the questionnaire on the second postpartum day during their stay in hospital. Each patient was informed about the aim of the study and gave their written consent to participate in the study. The questionnaires were delivered and collected by the main investigator.

### 2.2. Participants

The study group included adult pregnant women beyond the 37th week of gestation with a single pregnancy who were admitted for vaginal delivery, for whom labor pain relief methods were used, and who gave consent to complete the survey.

Participants under the age of 18, with multiple pregnancy, disturbed verbal communication or inability to communicate in Polish, inability to assess pain according to the NRS (Numeric Rating Scale), or who were indicated for elective caesarean section were excluded from the study.

### 2.3. Instrument

The study was conducted using an interview questionnaire, which consisted of two parts. The first part included sociodemographic questions concerning age, place of residence, education level, participation in antenatal classes, types of previous deliveries, sources of information about methods of labor pain relief, and a subjective determination of the pain threshold. The second part of the questionnaire contained questions regarding preferences and the efficacy of particular methods of labor pain relief, as well as the satisfaction of the women from the methods used during the labor. Method effectiveness was assessed by patients according to NRS before and after using the method. NRS ranged from 0 to 10, where NRS 0 = no pain and NRS 10 = unimaginable pain (mild pain—NRS 1–3, moderate pain—NRS 4–6, severe pain—NRS ≥ 7) [7]. Women’s satisfaction was measured with a 5-point Likert scale.

### 2.4. Data Collection

Data collection included: sociodemographic, pain threshold (minimal intensity of the stimulus that is perceived as painful), pain intensity scores, type of pain relief method, levels of reference of the hospital.

The methods of relieving labor pain were divided into two main categories: non-pharmacological and pharmacological methods. The non-pharmacological methods included physical activity (walking, assuming a position of comfort, moving), use of amenities (ball, bag, bean bag, ladder), massage of lumbosacral region, water immersion (shower, bathtub), and transcutaneous electrostimulation (Transcutaneous Electrical Nerve Stimulation—TENS). The pharmacological methods of treatment included nitrous oxide, epidural analgesia, opioid analgesics. The above-mentioned methods of relieving labor pain are most commonly used in Polish hospitals.

In Poland, there are three levels of hospital referentiality. Level I reference hospitals are centers for women in whom pregnancy develops properly and who deliver full-term babies. Such departments are prepared to take care of full-term neonates and those born between the 35th and 37th week of gestation, with neither significant pathologies nor symptoms of serious diseases. Level II reference hospitals are designated to take care of women in whom certain pathologies complicate the development of pregnancy. These hospitals must possess a neonatal intensive care unit. Level III hospitals provide the highest level of care, and this level refers to clinical hospitals. They admit women with high-risk pregnancies, demonstrating a pathological course and representing a high risk of delivering a preterm baby before completion of the 31st week of gestation or a child with various diseases and genetic defects.

Hospitals of all reference levels should have a spectrum of labor pain relief methods available according to the regulation of the Health Minister highlighted in the “Organizational Standard of Perinatal Care” [10].

### 2.5. Outcomes

The primary outcomes included the intensity of pain before and after using the non-pharmacological and/or pharmacological method for pain relief. The secondary outcomes were satisfaction of pain relief.

### 2.6. Sample Size Estimation

The basis for estimating the minimum size of the sample was the number of patients admitted to labor—in the year preceding the study—in hospitals where the study was conducted (*n* = 10,000). With a confidence level of 95%, a structure ratio of 50% and a assumed maximum error of 5%, the cohort should consist of 370 people.

### 2.7. Statistics

Qualitative variables were presented as numbers and percentages, while quantitative variables were characterized as median and upper and lower quartile. Non-parametric tests were used because the distribution of all quantitative variables departed from normality (variables were tested for normal distribution by the Shapiro–Wilk test). Differences between two dependent samples were checked with Wilcoxon probability test, while the Mann-Whitney U-test was used to test the independent samples. The Kruskal-Wallis test was applied to compare three independent samples (post hoc comparisons were computed). The correlation between numerical values was identified using the Spearman’s (R) rank correlation coefficient. Multivariable linear regression models were developed in order to identify the correlation between the satisfaction score (dependent variable) and independent variables such as: sociodemographic factors, pain threshold, median pain intensity before using the method/methods, type and number of pain relief method, levels of reference of hospital, response of staff to patient pain, informing the patient about methods of pain relief, possibility to choose the method of pain relief by the patient, and effectiveness of the methods used to relieve pain. In order to prepare the best multivariable model, a stepwise forward approach (equal probability of entry and removal was 0.05) was used, with selected independent variables based on simple linear regression models. Variables with a *p* value of less than 0.05 in simple linear regression models were entered into progressive stepwise regression. For each predictor, a standardized regression coefficient and a 95% confidence interval (CI) were determined. All statistical calculations were carried out using the Statistica 13.3. software (StatSoft, Poland).

## 3. Results

### 3.1. Patient Characteristics

A total of 500 women qualified for vaginal delivery took part in the study. Ultimately, the analysis included 404 women aged 18–40 hospitalized in five hospitals of the Pomeranian Voivodeship, an 80.8% response rate (Table 1). The largest group of respondents were women aged 24–30 (39.8%), with higher education (49%), working (81.4%) and city dwellers (68.5%). The median number of deliveries was 2 (1; 2). In the study group, 324 (80.2%) women gave birth by force of nature, while 80 (19.8%) underwent caesarean section. A total of 311 (77%) women declared the presence of an accompanying person during labor. The sources of knowledge for patients about the methods of relieving labor pain were: the Internet (*n* = 246; 60.9%), midwives (*n* = 197; 48.7%), birth schools (*n* = 180; 44.5%), opinions of family/friends (*n* = 144; 35.6%), obstetrician-gynecologist (*n* = 120; 29.7%), specialist literature (*n* = 58; 14.3%), anesthesiologist (*n* = 18; 4.4%). The respondents planned to use pain relief methods during labor such as: physical activity (*n* = 201; 49.7%), amenities (*n* = 198; 49.0%), epidural anesthesia (*n* = 177; 43.8%), water immersion (*n* = 113; 28.0%), TENS (*n* = 34; 8.4%).

### 3.2. The Intensity of Labor Pain in the Study Group

The intensity of labor pain, before the implementation of the methods of its alleviation, was assessed by the patients mainly as severe (*n* = 291; 72%), and for 5.2% of patients the pain was “unbearable” (NRS = 10). Every fourth patient experienced moderate pain (*n* = 103; 25.5%). Mild pain was declared by only 2.5% of patients (*n* = 10). Before implementing non-pharmacological and/or pharmacological methods the median severity of pain was 7 (6; 8), and after their application was 4.5 (3.5; 5.7); the inter-group difference was statistically significant (Z = 16.25; *p* < 0.01). The median of pain threshold values was 6 (4; 7)). There were no significant correlations between the pain threshold values and pain intensity before and after the implementation of non-pharmacological and/or pharmacological methods (*p* > 0.05)

### 3.3. Methods of Relieving Labor Pain in the Study Group

The detailed characteristics of the pain relief methods are presented in Table 2. Only non-pharmacological pain relief techniques were used in 161 (41.6%) patients, while in 236 (58.4%) patients mixed strategies (non-pharmacological and pharmacological) were implemented. The median number of pain relief methods used in each patient was 3 (2; 4). The use of both non-pharmacological techniques (median 6.7 (5; 8) vs. 4.5 (3.3; 5.5); Z = 15.02; *p* < 0.01) and pharmacological techniques (median 8 (7; 9) vs. 5 (3; 6); Z = 13.06; *p* < 0.01) resulted in a significant reduction in pain. Epidural anesthesia was the most effective method, while physical activity was the least effective. The patients declared that they had been informed about the available pain relief methods and rated this aspect of care highly (median 5 (4; 5)), as did the positive response of the medical staff to their pain (median 5 (4; 5)). On the other hand, they rated the availability of a choice of pain relief methods (median 4 (3; 5)) and their effectiveness (median 4 (3; 5)) lower.

### 3.4. The Intensity of Labor Pain and the Methods of Its Alleviation Depending on the Level of Hospital Reference

The intensity of pain before the implementation of pain relief methods was similar in all patients, regardless of the level of hospital reference, i.e., I, II, III (median 7.3 (5.7; 8.1) vs. 6.7 (5.6; 7.8) vs. 7 (6.2; 8); H = 4.79; *p* = 0.0909). Patients also had a similar level of pain threshold (median 5 (3; 7) vs. 6 (4; 8) vs. 6 (4; 7); H = 2.17; 0.3377). The effectiveness of the analgesic treatment depended on the hospital’s referentiality (H = 26.45; *p* < 0.0001). Post hoc comparisons showed that the median pain intensity in hospitals with reference grade I was significantly higher than in the hospital with reference grade II (5 (4; 6) vs. 4.2 (3; 5); Z = 4.16; *p* < 0.0001) and referential III (5 (4; 6) vs. 4.2 (3.3; 5.4); Z = 4.51; *p* < 0.0001). In hospitals of reference I, analgesic management was based primarily on the use of non-pharmacological techniques, less often mixed, i.e., non-pharmacological and pharmacological techniques (27.5%). On the other hand, in hospitals with higher referentiality, mixed methods were used more often (level II—65.8%; level III—81.2%). Epidural anesthesia was most often performed in the hospital of reference II (60%), and nitrous oxide was administered to patients in the hospitals of reference III (60.8%). The type of the applied methods of relieving labor pain depending on the reference of the hospital is presented in Table 3.

### 3.5. Predictors of Patient Satisfaction with Relieving Labor Pain

According to the Likert scale, the median satisfaction with the applied analgesic treatment was in the entire study group 4 (3; 5). The linear regression model for the satisfaction was statistically significant (*p* < 0.01) and very well fitted—the coefficient of determination was 63% (Table 4). The positive response of medical staff to patient pain, informing the patient about pain relief methods, possibility to choose the method of pain relief by the patient, and the effectiveness of the methods used to relieve pain have a positive impact on the satisfaction score. No use of TENS (negative regression coefficient) gave lower scores of satisfaction than using TENS.

## 4. Discussion

The results of the study showed that both non-pharmacological and pharmacological methods resulted in a significant reduction in pain. However, women giving birth in hospitals of higher referentiality have better control of labor pain due to access to pharmacological methods. Epidural anesthesia proved to be the most effective pain relief method.

An adequate understanding of the factors associated with labor pain paves the way for further pain acceptance by women and the effective use of pharmacological and non-pharmacological interventions for pain relief [11].

The intensity of experienced pain varies among women in labor, as some do not feel severe pain, while others describe their pain as the worst a woman may experience in her entire life. It should be noted, however, that labor pain is a unique type of pain, which does not represent any pathology [12,13].

The results of studies carried out by Najafi-Sharjabad et al. indicate that 45% of pregnant women chose vaginal delivery, while 41.1% opted for a caesarean section. The most frequent reasons for choosing a caesarean section and vaginal delivery were the fear of labor pain and a lower complication rate, respectively [14]. We attempted to find a potential correlation between patients with a high pain threshold and the selection of pharmacological methods of pain relief. Epidural analgesia was statistically significantly more often used in patients for whom the NRS score for the pain threshold exceeded 6. This may indicate that patients with a lower pain threshold are afraid not only of labor pain, but also of epidural analgesia, the procedure itself, and associated complications. Epidural analgesia or its combination with subarachnoid analgesia clearly gives a higher degree of labor pain relief when compared with parenteral opioids [4]. This correlation was demonstrated in the above-mentioned studies as the respondents found epidural analgesia to be the most efficient pharmacological method of pain relief.

The most commonly selected pharmacological method of pain relief in the study group was nitrous oxide (N_2_O). It is commonly used as an analgesic in many Western countries [15]. It was first used to reduce pain during labor by Stanislav Klikovich in Poland in 1881. Klikovich published the results of his studies, which included a group of 25 women in labor who were given a combination of 80% nitrous oxide and 20% oxygen. He observed pain relief with no adverse reaction in the fetus [15,16]. An analysis of the results of our study indicates that the highest analgetic efficacy was observed for epidural analgesia. Similar results were obtained by Czech et al. The mean pain scores in the first, second, and third phases of labor were 6.81 ± 2.26, 7.86 ± 2.06, and 3.22 ± 2.46, respectively. No significant difference in the pain intensity score was observed between groups receiving epidural and inhalation analgesia in the first phase of labor. Nevertheless, epidural analgesia reduced pain intensity in the second and third phases [17]. A review by Gill et al. demonstrated that women who took analgesics during labor more often experienced negative adverse effects, negative contacts with healthcare employees, and feelings of guilt or failure. However, the results of the above-mentioned studies did not demonstrate a reduction in the satisfaction of women using pharmacological methods of pain relief during delivery [18].

A research review by Anim-Somuah et al. pointed out that epidural anesthesia may be more effective in reducing pain during labor and increasing maternal pain satisfaction than non-epidural methods. While it generally appears that women experience an increase in assisted vaginal delivery with epidural anesthesia, a post hoc subgroup analysis has shown that this effect has not been seen in recent studies, most likely because of modern approaches to epidural analgesia in labor. Epidural analgesia had no effect on the risk of caesarean section or long-term back pain and did not appear to have a direct effect on the neonate’s condition as assessed by Apgar Scale or admission to neonatal intensive care units [19]. According to the researchers, the preferences of the delivery partner had an influence on the choice of the method of relieving labor pain and the possibility of using regional anesthesia. Research has also shown that a significant proportion of women who do not plan to use regional anesthesia during childbirth choose it nevertheless. This study also confirmed the association of socio-economic and ethnic factors with the use of epidural anesthesia during labor [20].

The most frequently used non-pharmacological method of pain relief in the studied group of patients was physical activity (343, 84.9). The highest analgesic efficacy, according to the respondents, was observed with the use of water immersion, and the least effective with the TENS method. A systematic review by Mascarenhas et al. included a total of 19 studies carried out between 2013 and 2018. The authors concluded that non-pharmacological methods such as acupuncture and its basic forms (acupressure and auriculotherapy) were chosen for pain relief by 29.17% of women, followed by hydrotherapy (25%), exercises with a ball (16.67%), the use of heat and cold (8.33%), and other methods (20.83%) [21]. Pilewska-Kozak et al. used non-pharmacological methods of pain relief and a 10-point scale to assess pain intensity before and after their use. The respondents declared massage to be the most effective (50, 32.5%), while vertical positions were the least often selected (10, 9.1%) [22]. Reports published by the World Health Organization demonstrate that natural techniques are most commonly used for prophylactic and protective reasons [23], while the results of the studies show that water immersion turned out to be the most effective non-pharmacological method for reducing labor pain.

In a randomized review of studies on the use and non-use of anesthesia in labor by Anim-Somuah M. et al., 38 studies were included with a total of 9658 women showing that epidural anesthesia brings the greatest satisfaction to the respondent [19]. They also confirm their own hypothesis, in which the method of anesthesia is the most effective in relieving the pain of labor.

Research by Harkins J. et al. shows that a significant number of women who do not want an epidural before birth get it during labor. This study also confirmed the association of socio-economic and ethnic factors with epidural use [6]. The research showed that respondents with higher education planned to take advantage of the start immersion, while the number of delivered births did not concern the choice of a specific method of delivery.

According to the research conducted by Lindholm A. et al., the most preferred methods of pain relief were also the most frequently used methods of pain relief; nitrous oxide, bath, breathing techniques, epidural, and massage. Women who used an epidural, regardless of their preference, were two to four times more likely to experience less positive birth experiences. Preference and priority were the most important factors in the actual use of painkillers. The epidural has been associated with less positive birth experiences [8]. On the other hand, in the authors’ own research, the most frequently chosen non-pharmacological method was water immersion and physical activity, while the most frequently chosen pharmacological method was nitrous oxide and epidural anesthesia.

Adequate pain relief is not always associated with the experience of high satisfaction by a mother, but understanding the opinion of women during labor is important for providing good quality postpartum care [18]. Prepartum education may prove helpful in obtaining more knowledge and having more realistic expectations about the course of delivery and more frequent use of non-pharmacological techniques of pain relief [14]. Some studies indicate that adequately early preparation is necessary to obtain a positive approach of women to labor and a more common use of non-pharmacological pain relief techniques. Our results show that water immersion was the most effective among non-pharmacological methods of pain relief. The study by Czech et al. also found that the highest level of satisfaction was observed among respondents with whom hydrotherapy was used [17]. Moreover, a study by Borodij et al. demonstrated that water immersion may successfully be used in patients with contraindications to epidural analgesia [24].

According to the respondents, the primary source of information regarding methods of labor pain relief was the Internet, while 255 subjects (25.6%) of the study group attended antenatal classes. Similar results were obtained by Pilewska-Kozak et al., whose patients obtained the greatest amount of information from websites, blogs, internet forums, social sites, and groups run by midwives or obstetricians, but not from direct consultations with healthcare professionals. Only 40 patients (35.7%) attended antenatal classes [22].

The analysis of our results shows that epidural anesthesia is the most effective among pharmacological methods. These results were also confirmed in the studies by Czech et al. [17]. Epidural is the most effective, but it has no impact on satisfaction in our study. Satisfaction was most influenced by: positive response of staff to patient pain, informing the patient about methods of pain relief, possibility to choose the method of pain relief by the patient, and effectiveness of the methods used to relieve pain have a positive impact on the satisfaction score. No use of TENS (negative regression coefficient) gave lower scores of satisfaction than using TENS. In the studies of Suarez et al. it was shown that the highest level of satisfaction was presented by women who received TENS [25]. In the studies by Gönenç et al. It was found that the use of massage significantly reduced the perceived pain of labor in all stages of labor, while the acupressure intervention significantly reduced the perceived pain of labor only during the active and transitional phase of labor. Both interventions successfully increased maternal satisfaction [26]. In the studies by Tan et al. it has been shown that patients who underwent epidural catheter placement had a higher risk of dissatisfaction. However, the authors stated that further research was needed [27]. Hodnett, on the other hand, found in a systematic review of women’s pain and childbirth satisfaction that the effects of pain, pain relief, and medical interventions during childbirth on subsequent satisfaction are not as obvious, direct, or as strong as the effects of caregivers’ attitudes and behavior [28].

Further studies are needed. The availability of methods depends on the degree of reference, and the availability of equipment and qualified personnel. The reported observations indicate that non-pharmacological techniques, even when combined with pharmacological methods, may be beneficial for women. Satisfaction from the delivery depends not only on the level of experienced pain but also on the care received during pregnancy and labor. Efficient pain management has become such an important part of the care plan for women of child-bearing age that it was included in the Organizational Standard of Peripartum Care issued by the Ministry of Health. This publication is an index document for hospitals regarding such issues as the use and availability of labor pain relief methods. Pregnant women should be educated on the available methods of pain relief during delivery and receive adequate assistance in coping with labor-related fears [10].

## 5. Limitations

A limitation in the study was the lack of availability of all the methods used to relieve labor pain. In hospitals with a lower degree of referentiality, non-pharmacological and pharmacological methods are most often used, specifically those that do not require the involvement of an anesthesiologist.

## 6. Conclusions

The most commonly selected techniques of labor pain relief by pregnant women in level I referral hospitals and level II referral hospitals were non-pharmacological (physical activity, use of amenities, water immersion) and pharmacological methods (nitrous oxide, epidural analgesia), respectively. Water immersion and epidural analgesia turned out to be the most efficient among non-pharmacological and pharmacological methods, respectively. Epidural anesthesia remains the most effective pharmacological method for relieving labor pain. The factors determining the choice of a specific method are determined by the degree of hospital referentiality.

## 7. Implications for Obstetrician Practice

Own research has shown that the Internet is still the most frequently used source of knowledge. Unfortunately, the information contained therein is not always reliable, there-fore the knowledge should be more widely popularized by specialists working, among others, in birthing schools. The degree of success of the pain relief technique used in hospitals depends on the availability of maternity staff. Although healthcare professionals do not need to be specialized in the subject of alternative pain relief therapies for labor, they should have a basic understanding of the subject and should be able to advise women on further information, as the need for alternative treatments in obstetrics is likely to increase. It is also worthwhile, due to the effectiveness of epidural anesthesia demonstrated in studies, to strive to secure the Delivery Wards with appropriately qualified personnel, so that the availability of anesthesia is as high as possible, irrespective of hospital referentiality.

## Figures and Tables

**Table 1:** Sociodemographic characteristics of subjects (*n* = 404).

Variables	*n* (%)
Age (years)	
18–23	51 (12.6)
24–30	161 (39.8)
30–34	136 (33.7)
35–40	56 (13.9)
Education	
University	198 (49.0)
High school	147 (36.4)
Vocational education	45 (11.1)
Primary school	14 (3.5)
Employment status	
Employed	329 (81.4)
Unemployed	75 (18.5)
Place of residence	
City up to 100,000 inhabitants	97 (24.0)
City from 100,000–250,000 inhabitants	44 (11.0)
City over 250,000 inhabitants	136 (33.7)
Village	127 (31.4)
Number of births	
1	174 (43.1)
2	153 (37.9)
3	51 (12.6)
4	20 (4.9)
5	2 (0.5)
6	2 (0.5)
7	2 (0.5)
Level of hospital reference	
I	149 (36.9)
II	79 (19.5)
III	176 (43.5)

Categorical data were reported as number and percentage.

**Table 2:** Pain intensity (in NRS) before and after using the methods for pain relief.

Methods	*n*	NRS before Using the Method	NRS after Using the Method	*z* Value	*p* Value
Non-pharmacological	384	6.7 (5; 8)	4.5 (3.3; 5.5)	14.63	<0.01
Physical activity	343	6 (5; 8)	5 (3; 6)	12.69	<0.0001
Use of amenities	267	7 (5; 8)	5 (3; 6)	11.04	<0.0001
Massage of lumbosacral region	161	7 (5; 9)	5 (4; 6)	9.14	<0.0001
Water immersion	221	7 (6; 8)	5 (3; 6)	10.98	<0.0001
TENS	58	7 (5; 8)	5 (4; 6)	5.32	<0.0001
Pharmacological	256	8 (7; 9)	5 (3; 6)	13.05	<0.01
Nitrous oxide	172	8 (7; 9)	6 (5; 7)	9.69	<0.0001
Epidural analgesia	128	8 (7; 10)	3 (2; 4)	9.56	<0.0001
Opioid analgesics	64	7 (5; 9)	5 (3; 6)	5.63	<0.0001

Descriptive statistics are expressed as a median and upper and lower quartile, NRS—Numeric Rating Scale.

**Table 3:** The level of hospital referentiality as a determinant of the choice of the pain relief method.

Variables	The Level of Hospital Referentiality			χ2 Value	*p* Value
	I (*n* = 149)	II (*n* = 79)	III (*n* = 176)		
Non-pharmacological methods	149 (100.0)	67 (84.8)	168 (95.4)	25.42	<0.0001
Physical activity	139 (93.3)	66 (83.5)	138 (78.4)	14.08	0.0008
Use of amenities	124 (83.2)	34 (43.0)	109 (61.9)	39.6	<0.0001
Massage of lumbosacral region	82 (55.0)	32 (40.5)	47 (26.7)	27.03	<0.0001
Water immersion	108 (72.5)	26 (32.9)	87 (49.4)	36.12	<0.0001
TENS	32 (21.5)	15 (19.0)	11 (6.2)	16.92	0.0002
Pharmacological methods	41 (27.5)	64 (81.0)	151 (85.8)	131.23	<0.0001
Nitrous oxide	30 (20.1)	35 (44.3)	107 (60.8)	54.69	<0.0001
Epidural analgesia	13 (8.7)	45 (60.0)	70 (39.8)	64.93	<0.0001
Opioid analgesics	25 (16.8)	25 (31.6)	14 (7.9)	23.11	0.00001
Mixed methods	41 (27.5)	52 (65.8)	143 (81.2)	98.12	<0.0001

Categorical data were reported as number and percentage; mixed methods: non-pharmacological and pharmacological.

**Table 4:** The results of regression analysis of predictors of the satisfaction of pain relief.

Predictors	Simple Regression Beta (95% CI)	Multivariable Regression	
		Beta (95% CI)	Model
Positive response of staff to patient pain	0.53 (0.45 to 0.62) ***	0.21 (0.13 to 0.28) ***	R^2^ = 0.63; df = 5 F Value = 139.58 *p* < 0.01
Informing the patient about methods of pain relief	0.57 (0.49 to 0.65) ***	0.15 (0.06 to 0.24) ***	
Possibility to choose the method of pain relief by the patient	0.57 (0.49 to 0.65) *	0.15 (0.06 to 0.23) **	
Effectiveness of the methods used to relieve pain	0.70 (0.63 to 0.77) *	0.49 (0.42 to 0.56) ***	
TENS ^No vs. Yes^	−0.15 (−0.25 to −0.06) *	-0.07 (-0.13 to -0.01) ***	
Place of residence	0.10 (0.01 to −0.20) *		
Epidural analgesia	−0.22 (−0.32 to −0.12) ***		
Hospital referentiality ^II vs. III^	0.18 (0.06 to 0.31) *		
Number of pain relief methods used	0.11 (0.02 to 0.21) *		

* *p* < 0.05; ** *p* < 0.001; *** *p* < 0.0001; Beta—standardized regression coefficient; R2—adjusted coefficient of determination; CI—confidence interval.

## Data Availability

A dataset will be made available upon request to the corresponding authors one year after the publication of this study. The request must include a statistical analysis plan.
